# Glutaminase-2 Expression Induces Metabolic Changes and Regulates Pyruvate Dehydrogenase Activity in Glioblastoma Cells

**DOI:** 10.3390/ijms26010427

**Published:** 2025-01-06

**Authors:** Juan De los Santos-Jiménez, José A. Campos-Sandoval, Tracy Rosales, Bookyung Ko, Francisco J. Alonso, Javier Márquez, Ralph J. DeBerardinis, José M. Matés

**Affiliations:** 1 Departamento de Biología Molecular y Bioquímica, Universidad de Málaga, 29071 Málaga, Spain; jacs@uma.es (J.A.C.-S.); fcarrion@uma.es (F.J.A.); marquez@uma.es (J.M.); 2 Instituto de Investigación Biomédica de Málaga (IBIMA-Plataforma BIONAND), Universidad de Málaga, 29071 Málaga, Spain; 3 Children’s Medical Center Research Institute, University of Texas Southwestern Medical Center (UTSMC), Dallas, TX 75390, USA; tracyrsls@gmail.com (T.R.); bookyung.ko@utsouthwestern.edu (B.K.); ralph.deberardinis@utsouthwestern.edu (R.J.D.); 4 McDermott Center for Human Growth and Development, University of Texas Southwestern Medical Center (UTSMC), Dallas, TX 75390, USA; 5 Department of Pediatrics, University of Texas Southwestern Medical Center (UTSMC), Dallas, TX 75390, USA

**Keywords:** cancer, glioblastoma, glutamine, glutaminase, glutaminase-2, metabolomics, pyruvate dehydrogenase

## Abstract

Glutaminase controls the first step in glutaminolysis, impacting bioenergetics, biosynthesis and oxidative stress. Two isoenzymes exist in humans, GLS and GLS2. GLS is considered prooncogenic and overexpressed in many tumours, while GLS2 may act as prooncogenic or as a tumour suppressor. Glioblastoma cells usually lack GLS2 while they express high GLS. We investigated how GLS2 expression modifies the metabolism of glioblastoma cells, looking for changes that may explain GLS2’s potential tumour suppressive role. We developed LN-229 glioblastoma cells stably expressing GLS2 and performed isotope tracing using U-^13^C-glutamine and metabolomic quantification to analyze metabolic changes. Treatment with GLS inhibitor CB-839 was also included to concomitantly inhibit endogenous GLS. GLS2 overexpression resulted in extensive metabolic changes, altering the TCA cycle by upregulating part of the cycle but blocking the synthesis of the 6-carbon intermediates from acetyl-CoA. Expression of GLS2 caused downregulation of PDH activity through phosphorylation of S293 of PDHA1. GLS2 also altered nucleotide levels and induced the accumulation of methylated metabolites and S-adenosyl methionine. These changes suggest that GLS2 may be a key regulator linking glutamine and glucose metabolism, also impacting nucleotides and epigenetics. Future research should ascertain the mechanisms involved and the generalizability of these findings in cancer or physiological conditions.

## 1. Introduction

The reprogramming of cellular metabolism is a hallmark of cancer [1]. Tumours present metabolic adaptations that allow them to meet the increased need for energy generation, the biosynthesis of new cell compounds and the maintenance of biochemical homeostasis [2]. Although the concept of metabolic reprogramming is built upon the notion that cancer cells possess a plethora of metabolic adaptations that increase their fitness, it may also render them vulnerable to specific targeted therapies against these metabolic traits, which are fundamental for their survival and success [3,4]. Glutamine (Gln) provides a large reservoir for nitrogen in animal cells [5] and is by far the most abundant amino acid in human plasma [6]. The importance of Gln in cancer metabolism has been established in seminal articles [6,7]. In cancer, Gln may constitute an essential nutrient, fuelling bioenergetics and serving to obtain ATP, promoting the biosynthesis of non-essential amino acids [8], nucleotides [9], lipids [10] hexosamines, and also being linked to the synthesis of the critical antioxidant glutathione (GSH) [11]. Therefore, Gln constitutes a source of both reduced nitrogen and carbon and is a main anaplerotic substrate in cancer cells, being the principal supplier for the tricarboxylic acid (TCA) cycle in cultured cells in vitro [12].

Gliomas account for the majority of tumours arising from the central nervous system [13]. Although glycolysis provides energy and carbon skeletons for the synthesis of amino acids, lipids and nucleotides in gliomas, glutaminolysis and oxidative phosphorylation (OXPHOS) are also critical for their metabolism [14]. Among gliomas, glioblastoma multiforme (GBM) is the most common and lethal, showing a very low 5-year survival rate [15]. Current treatment of GBM mainly consists of maximal surgical resection followed by radiotherapy and chemotherapy using the alkylating agent temozolomide; however, the efficacy is limited, as median survival time ranges from 12 to 15 months, so new therapeutic options are needed [16]. Gliomas, including GBM, are classified as isocitrate dehydrogenase (IDH)-mutant or IDH-wild type based on whether they harbour mutations in IDH1 or IDH2 [17]. Common mutations in IDH1 or IDH2 confer a gain of function, resulting in a non-canonical enzymatic activity converting α-ketoglutarate (AKG) into the oncometabolite D-2-hydroxyglutarate (D-2HG), which interferes with dioxygenase function leading to a prooncogenic hypermethylation phenotype [18]. Other common genetic alterations in GBMs include the gain of function of the epidermal growth factor receptor (EGFR) [19], or the inactivation of mutations in tumour suppressors such as the phosphatase and tensing homolog (PTEN), CDKN2A, or TP53 [15]. Gliomas and GBMs usually reprogram Gln metabolism, as tumours become avid consumers of Gln [20]. In this regard, it is common the upregulation of glutaminase [21].

Glutaminase (GA; EC 3.5.1.2) is the enzyme responsible for catalyzing the oxidative deamidation of Gln, producing glutamate and free ammonium in the first step in glutaminolysis [11,22]. Although there are many other essential enzymes employing Gln as a substrate and producing glutamate, as Gln amidotransferases, their action implies the transfer of the amide group of Gln to an acceptor molecule [23]. Cancer cells usually show much greater rates of ammonium generation and excretion compared to ammonium incorporation into biomass [24]. As the ammonium incorporation rate may be limiting the production of glutamate from Gln by these enzymes, GAs become principal enzymes for tumour cells, allowing for faster Gln-metabolizing rates [25]. Accordingly, GAs’ expression patterns are commonly altered in cancer [26]. In humans, two genes encode for GAs, *GLS* and *GLS2*. *GLS* encodes two isoforms, the larger kidney-type glutaminase (KGA) and the shorter glutaminase C (GAC), and both can be called indistinctly GLS isoforms. On the other hand, the *GLS2* gene encodes for a canonical isoform, glutaminase B (GAB), and a shorter one called liver-type glutaminase (LGA), being both collectively named as GLS2 isoforms [27]. Expression of GLS is favoured by oncogenes such as c-Myc [28], while GLS2 was found to be a direct target of p53 [29]. Many tumours, including GBMs, overexpress the GLS isoenzymes, which correlates with malignancy, and therefore GLS isoforms have been considered prooncogenic factors [30]. Several GLS inhibitors have been investigated for potential application in therapy, including compound CB-839, which reached clinical trials [17,26,31]. On the other hand, GLS2 is silenced in several cancer cell lines compared with the normal tissue of origin, as it takes place in hepatocellular carcinoma (HCC), colon carcinomas and GBMs, where *GLS2* promoter was found to be hypermethylated [32,33]. GLS inhibition by CB-839 induced cell cycle arrest in GLS-highly expressing GBM stem-like cells [34], while it slowed proliferation in T98-G and LN-229 GBM cell lines [35].

In this work, we investigated the metabolic changes upon restoring the expression of GLS2 in a GBM cell model, the human cell line LN-229, characterized by no detectable GLS2 expression but expressing GLS at high levels [36]. We developed cell models with stable overexpression of GLS2 and conducted a broad investigation of cellular metabolism including metabolomics and stable isotope tracing to study how GBM cell metabolism changes before and after GLS2 overexpression. We also included concomitant treatment with the GLS inhibitor CB-839, looking to distinguish isoenzyme-specific metabolic changes. Our results showed that GLS2 induced broad metabolic changes in GBM cells, affecting metabolites related to the TCA cycle, nucleotides and epigenetic-related metabolites, suggesting that GLS2 has a key role connecting Gln and glucose metabolism, and potentially affecting bioenergetics. Key questions to be solved include deeper mechanistic insights and how generalizable these GLS2-induced changes are in other tumour types and in organs expressing GLS2 in physiological conditions, as it happens in the liver. Furthermore, understanding these roles may pave the way for identifying specific context-dependent molecular vulnerabilities to be exploited in future therapeutic approaches.

## 2. Results

### 2.1. GLS2 Overexpression Slows Cell Proliferation in Human LN-229 Cell Line and Confers Resistance to CB-839 Treatment

**Figure 1:**
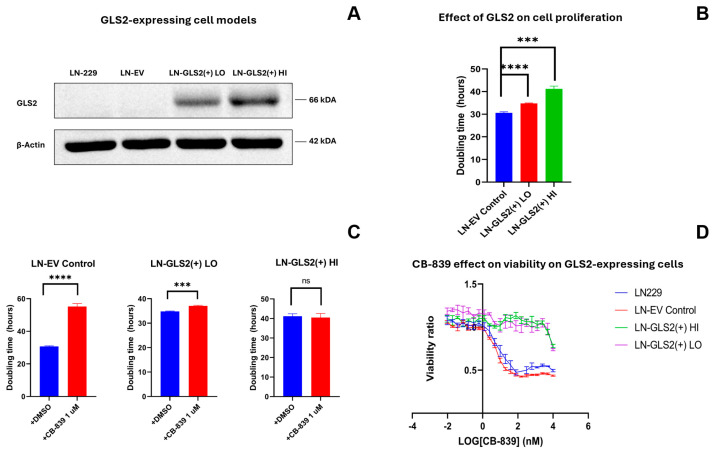
Effect of GLS2 expression on cell proliferation and CB-839 susceptibility. (**A**) A representative Western blot of the cell models employed, showing GLS2 overexpression for the two models expressing GLS2 at different levels, named LN-GLS2(+) LO for the lower expressing model, and LN-GLS2(+) HI, for the higher expressing model, and compared to the wild-type LN-229 cells and the sham-transfected empty vector cells (LN-EV). (**B**) Mean doubling time of the two GLS2-overexpressing models compared to the LN-EV control; higher GLS2 expression correlates with a significantly higher doubling time. (**C**) Comparison of the mean doubling time for each experimental condition when CB-839 is added at a concentration of 1 µM. LN-EV control cells significantly increased their doubling time almost to double, while LN-GLS2(+) LO had only a slight increase, and no change was noted for LN-GLS2(+) HI. (**D**) Cell viability ratio of the GLS2-expressing models compared to the wild type and EV controls when CB-839 is added. Asterisks note the statistical significance of a *t*-test for these comparisons, as follows: ***: *p*-value < 0.001; ****: *p*-value < 0.0001; ns: non-significant. Western-blotting experiments were repeated three times. Cell proliferation was repeated five times, in triplicate. Cell viability was assessed in quadruplicate.

To investigate the effects of GLS2 expression on human GBM cells originally lacking GLS2, we developed two independent cell models stably expressing GLS2. LN-229 wild-type cells or cells transfected with the empty vector to be used as a control (LN-EV) showed no detectable expression of GLS2 protein, while the two selected models overexpressed GLS2 at a lower (called LN-GLS2(+) LO) or higher (called LN-GLS2(+) HI) level (Figure 1A). Cell proliferation was assessed to calculate the doubling time for each cell model (Figure 1B). The mean doubling time for LN-EV control was 30.6 h, while it increased to 34.8 h for LN-GLS2(+) LO, and to 41.2 h for LN-GLS2(+) HI. Thus, GLS2 expression increased the time required for cell duplication by 13.7% and 34.6%, respectively. When GLS inhibitor CB-839 was added at 1 µM, LN-EV control cells nearly doubled their doubling time compared to untreated cells (Figure 1C), while a slight increase (6.6%) was observed for LN-GLS2(+) LO, and no change was noted for LN-GLS2(+) HI. Additionally, a cell viability assay was performed employing a wide range of CB-839 concentration treatments for 72 h (Figure 1D). Increasing CB-839 concentration decreased the viability ratio of LN-229 and LN-EV to 50%, with calculated IC50s of 47.4 nM and 33.3 nM, respectively. GLS2-expressing cell models showed no significant decrease in viability.

### 2.2. GLS2 Alters Both Oxidative Decarboxylation and Reductive Carboxylation of Gln-Derived AKG in the TCA Cycle

Glutaminolysis is a main anaplerotic pathway in cancer cells. GAs convert Gln into glutamate, which may subsequently generate AKG, an intermediate metabolite of the TCA cycle. AKG normally follows the oxidative pathway to form succinyl-CoA and, subsequently, succinate, fumarate, malate and oxaloacetate, which may react with acetyl-CoA coming from glycolytic pyruvate to form citrate, and citrate may be isomerized to cis-aconitate and isocitrate, which can be decarboxylated to AKG by IDH, closing the cycle. Alternatively, cancer cells may activate an alternative pathway for AKG, implying its reductive carboxylation to isocitrate in a non-canonical reaction catalyzed by IDHs, which may serve to boost lipid biosynthesis from Gln. To analyze how Gln anaplerotic metabolism is affected by GLS2 expression, cell models were supplied with Gln isotopically labelled in all its carbons with ^13^C ([U-^13^C]Gln), treated or not treated with the GLS inhibitor CB-839 at 1 µM for 24 h, to account for an experimental condition in which GLS2 is being expressed while GLS activity is inhibited, so most GA activity would be due to GLS2. Metabolites generated from Gln carbon will inherit part or all of the ^13^C label. Thus, succinate, fumarate, malate and aspartate produced from Gln following the oxidative TCA cycle metabolism, will inherit the m + 4 label, while citrate generated by reductive carboxylation of AKG will be m + 5. Fumarate, malate or aspartate produced from reductive citrate will be m + 3.

**Figure 2:**
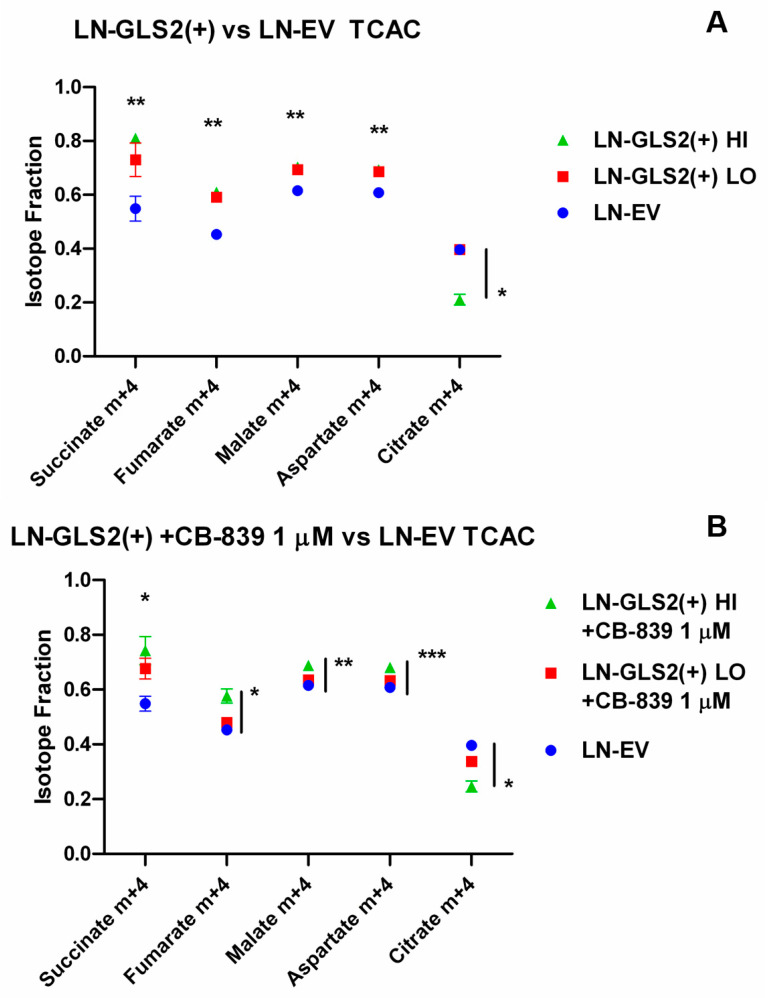
GLS2 alters carbon contributions from Gln to the TCA cycle. Isotopologue fractions of the total metabolite pool for selected metabolites related to the TCA cycle. The graph shows the m + 4 fractions of metabolites labelled from U-^13^C-Gln after 6 h. (**A**) M + 4 fractions of succinate, fumarate, malate and aspartate are significantly higher in both GLS2-overexpressing models, while citrate m + 4 is significantly diminished (50%) in LN-GLS2(+) HI. (**B**) When GLS2-overexpressing models were treated with CB-839 1 µM, m + 4 fractions of fumarate, malate and aspartate of the LN-GLS2(+) LO model returned to the levels of the untreated LN-EV control, while citrate m + 4 is still reduced in the LN-GLS2(+) HI model, not being affected by the CB-839 treatment. Asterisks note the statistical significance of a *t*-test for the comparison of the GLS2-expressing model vs. the LN-EV control, as follows: *: *p*-value < 0.05; **: *p*-value < 0.01; ***: *p*-value < 0.001. Asterisks above data indicate significance for the comparison of both LN-GLS2(+) models vs. LN-EV. When vertical bars are included, only statistical significance was found between LN-GLS2(+) HI and LN-EV. The experiment was made three times, each condition in triplicate.

Figure 2 shows the fractional abundances of m + 4 isotopologues from several metabolites related to the TCA cycle in the GLS2-overexpressing models compared to LN-EV. GLS2 expression increased the labelling from Gln in oxidatively generated succinate, fumarate, malate and aspartate (Figure 2A). Surprisingly, LN-GLS2(+) HI shows a 50% reduction in citrate m + 4 compared to LN-EV. When LN-GLS2(+) LO was treated with CB-839 (Figure 2B), fractional abundances of m + 4 fumarate, malate and aspartate returned to the control values. However, LN-GLS2(+) HI treated with CB-839 showed almost no change compared to untreated LN-GLS2(+) HI. Interestingly, m + 4 citrate reduction did not change with CB-839 treatment, showing the same differences against the LN-EV control.

**Figure 3:**
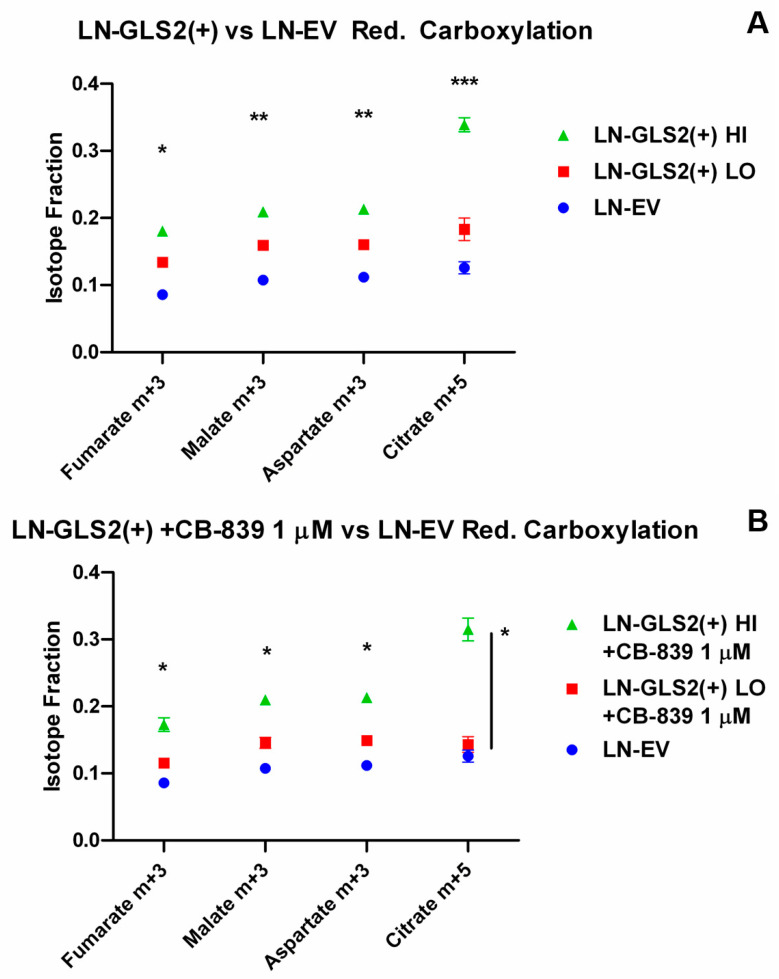
GLS2 impacts reductive carboxylation-associated labelling from Gln carbons. Isotopologue fractions of the total metabolite pool for selected metabolites related to reductive carboxylation of Gln-derived AKG. The graph shows the m + 5/m + 3 fractions of metabolites labelled from U-^13^C-Gln after 6 h. (**A**) Higher GLS2 expression (LN-GLS2(+) HI, green triangles) correlates to a large increase in citrate m + 5, as well as higher m + 3 labelling of fumarate, malate and aspartate. (**B**) Treatment with CB-839 1 µM caused no changes in isotopologue abundances compared to the untreated GLS2-expressing models from **A**. Asterisks note the statistical significance of a *t*-test for the comparison of the GLS2-expressing model vs. the LN-EV control, as follows: *: *p*-value < 0.05; **: *p*-value < 0.01; ***: *p*-value < 0.001. Asterisks above data indicate significance for the comparison of both LN-GLS2(+) models vs. LN-EV. When vertical bars are included, only statistical significance was found between LN-GLS2(+) HI and LN-EV. The experiment was made three times, each condition in triplicate.

Figure 3 shows fractional abundances for reductive carboxylation-associated labelling. Expression of GLS2 increased the labelling of m + 3 fumarate, malate and aspartate (Figure 3A), and in the case of LN-GLS2(+) HI, a large increase in m + 5 citrate (from 0.12 of LN-EV Control to 0.35). These changes only showed a slight reduction in LN-GLS2(+) LO when treated with CB-839 (Figure 3B), while treated LN-GLS2(+) HI showed no difference compared to the untreated model.

### 2.3. Effect of GLS2 Overexpression on the Metabolome

**Figure 4:**
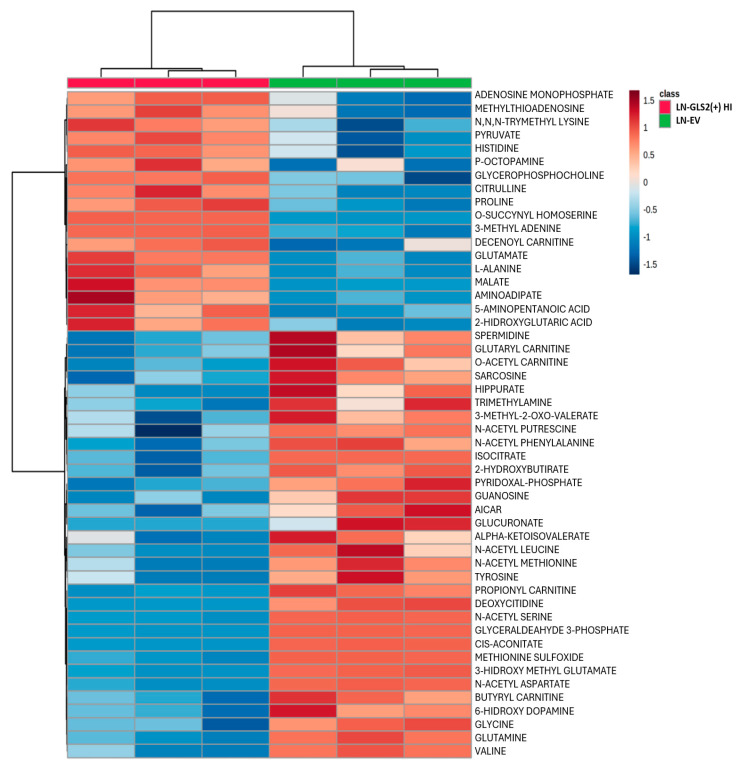
Metabolic changes induced by GLS2 expression. Metabolomic changes induced by GLS2 expression are depicted on a hierarchical clustering analysis of metabolites in the LN-229 cell line. The heatmap shows the change in abundance for the top 50 changing metabolites comparing the LN-GLS2(+) HI model to the LN-EV control. Two independent experiments were made, each condition in triplicate.

**Figure 5:**
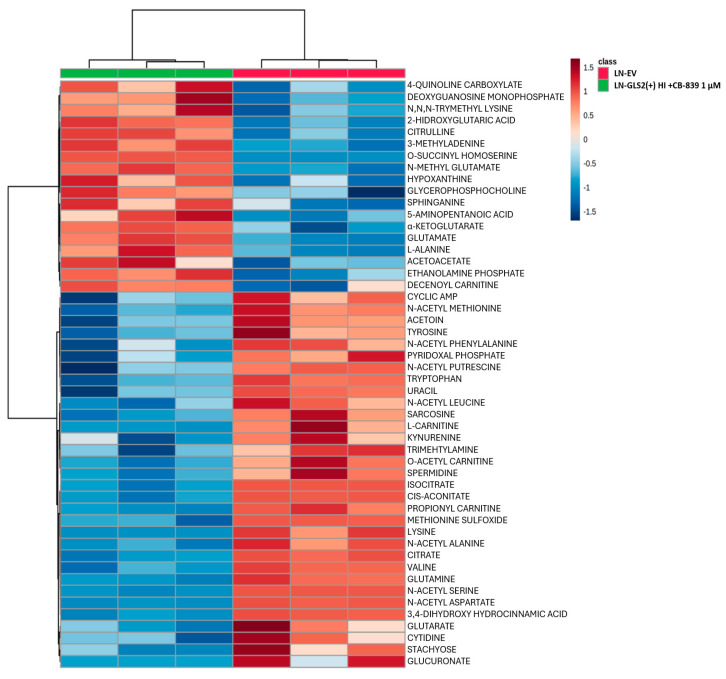
Metabolic changes induced by GLS2 expression and concomitant GLS activity inhibition. Metabolomic changes induced by the combination of GLS2 expression and concomitant treatment with the CB-839 inhibitor are depicted on a hierarchical clustering analysis of metabolites in the LN-229 cell line. The heatmap shows the change in abundance for the top 50 changing metabolites comparing the LN-GLS2(+) HI model + CB-839 1 µM to the LN-EV control. Two independent experiments were made, each condition in triplicate.

To further analyze the metabolic changes induced by GLS2 expression and by GLS2 expression and concomitant GLS inhibition by CB-839 (1 µM for 24 h), we carried out a broad LC–MS-based metabolomic analysis for the quantification of the levels of 306 metabolites. Of all the metabolites detected and quantified, more than 100 changed their levels significantly in the comparison between the two GLS2-expressing models and LN-EV. When CB-839 was added, the treated models showed differential amounts of more than 80 metabolites. A hierarchical clustering analysis was performed using the web tool Metaboanalyst. To provide a general view of the metabolic changes induced by GLS2, Figure 4 shows a heatmap for the change in abundance of the top 50 metabolites in the comparison between LN-GLS2(+) HI and LN-EV, while Figure 5 shows the top 50 changing metabolites in LN-GLS2(+) HI +CB-839 vs. LN-EV.

Among changes in the metabolome, GLS2 expression was found to affect the levels of several nucleotides (Appendix B). LN-GLS2(+) HI showed an increase of 2.3-fold for adenosine monophosphate (AMP), while guanosine monophosphate (GMP) and uridine monophosphate (UMP) levels dropped to 0.72 and 0.52-fold, respectively (Appendix B, panel A). LN-GLS2(+) LO showed an increase of 1.94-fold for AMP but showed no statistically significant change for GMP and UMP, although tending to drop (0.76 and 0.63, respectively) (Appendix B, panel B). When LN-GLS2(+) HI was treated with CB-839, the increase in AMP was slightly attenuated (1.88-fold vs. LN-EV), but GMP and UMP were reduced compared to the untreated model (0.64 and 0.40 vs. LN-EV, respectively) (Appendix B, panel C). LN-GLS2(+) LO + CB-839 still showed an accumulation of AMP (1.75-fold), and, interestingly, showed a significant drop in GMP and UMP (0.53 and 0.49-fold, respectively) (Appendix B, panel D).

GLS2 expression was also shown to alter the levels of two methylated metabolites, 3-methyl-adenine (3-MA) and N,N,N-trimethyl-lysine (Appendix C, panels A and B). LN-GLS2(+) LO caused 3-MA levels to rise to 1.6-fold, while higher GLS2 expression caused a further increment to 1.8-fold. Noticeably, treatment with CB-839 caused 3-MA levels to increase (Appendix C, panel A). Both GLS2 expression and GLS inhibition by CB-839 made 3-MA levels rise. The pattern was quite similar for N,N,N-trimethyl-lysine (Appendix C, panel B), which raised to 1.6 and 1.9-fold for untreated LN-GLS2(+) LO and HI models, respectively, and further increased when CB-839 was added.

Interestingly, the levels of the universal methyl donor, S-adenosyl methionine (SAM) were increased to 2.3-fold in LN-GLS2(+) HI, while this increase was not affected by CB-839 treatment (Appendix C, panel C).

However, GLS2 expression significantly affected glutaminolysis and the TCA cycle, and the metabolic changes related to these pathways will be presented more profusely in the following section.

### 2.4. GLS2 Reshapes Key Metabolic Pathways in Human GBM Cells

**Figure 6:**
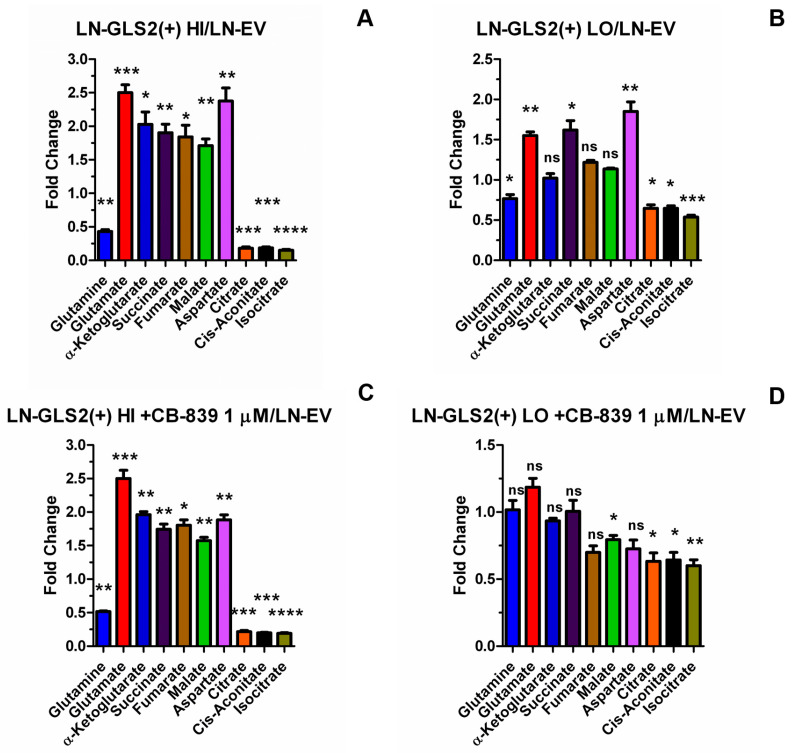
GLS2 remodels the TCA cycle. Fold-change for metabolites related to the TCA cycle, comparing LN-GLS2(+) HI vs. LN-EV (**A**), LN-GLS2(+) LO vs. LN-EV (**B**), LN-GLS2(+) HI + CB-839 1 µM vs. LN-EV (**C**) or LN-GLS2(+) LO + CB-839 1 µM vs. LN-EV (**D**). Asterisks note the statistical significance of a *t*-test for these comparisons, as follows: *: *p*-value < 0.05; **: *p*-value < 0.01; ***: *p*-value < 0.001; ****: *p*-value < 0.0001; ns: non-significant. Two independent experiments were made, each condition in triplicate.

GLS2 overexpression altered the levels of TCA cycle metabolites, as shown in Figure 6. LN-GLS2(+) HI showed lower levels of Gln (0.43-fold), while glutamate was higher (2.5-fold) (Figure 6A). Metabolites downstream of glutamate production by GAs also changed, as shown by the levels of AKG (2-fold), succinate (1.9-fold), fumarate (1.8-fold), malate (1.7-fold) and aspartate (2.4-fold). Notably, citrate (0.18-fold), cis-aconitate (0.18-fold) and isocitrate (0.15-fold) levels were strongly reduced in LN-GLS2(+) HI, exhibiting a drop of more than 80% compared to the control. LN-GLS2(+) LO showed smaller differences for these metabolites, proportional to GLS2 expression: Gln (0.77-fold), glutamate (1.6-fold), succinate (1.6-fold), aspartate (1.9-fold), citrate (0.65-fold), cis-aconitate (0.65-fold), and isocitrate (0.54-fold) (Figure 6B).

LN-GLS2(+) HI treated with CB-839 1 µM for 24 h showed no changes compared to the untreated condition (Figure 6C). When LN-GLS2(+) LO was treated with CB-839, the levels of Gln, glutamate, succinate and aspartate returned to the control ones, while malate levels were significantly lower (Figure 6D). Interestingly, the levels of citrate, cis-aconitate and isocitrate were still significantly lower, not being affected by CB-839 treatment.

**Figure 7:**
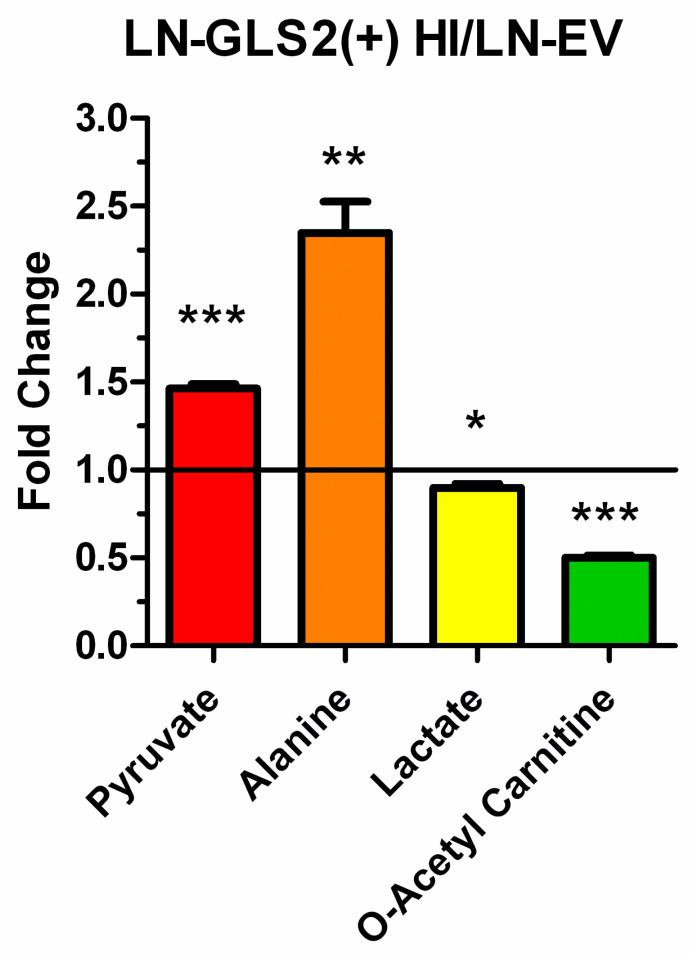
GLS2 modifies the levels of pyruvate and related metabolites. Fold-change for key metabolites related to glycolytic pyruvate funnelling to the tricarboxylic acid (TCA) cycle and pyruvate dehydrogenase (PDH) function, including pyruvate, alanine, lactate and O-acetyl carnitine (the latter, intended as a potential indicator of acetyl-CoA levels), comparing LN-GLS2(+) HI vs. LN-EV. Asterisks note the statistical significance of a *t*-test for these comparisons, as follows: *: *p*-value < 0.05; **: *p*-value < 0.01; ***: *p*-value < 0.001. Two independent experiments were made, each condition in triplicate.

LN-GLS2(+) HI also showed a significant 1.5-fold increase in pyruvate levels (Figure 7), and in L-alanine (2.4-fold). Interestingly, pyruvate accumulation did not correlate with an increase in lactate, which showed a slight but significant decrease when GLS2 was expressed at high levels (0.90-fold). Additionally, O-acetyl carnitine was significantly decreased to 0.50-fold.

In addition, the levels of several N-acetyl amino acids decreased when GLS2 was overexpressed, treated or not treated with CB-839 (Appendix D), as it took place in all cases for N-acetyl aspartate, N-acetyl glycine or N-acetyl serine. LN-GLS2(+) HI showed greater differences compared with the control, with a significant decrease in the levels of N-acetyl alanine (0.59-fold), N-acetyl asparagine (0.47-fold), N-acetyl aspartate (0.15-fold), N-acetyl cysteine (0.46-fold), N-acetyl glycine (0.35-fold), N-acetyl leucine (0.65-fold), N-acetyl methionine (0.68-fold), N-acetyl phenylalanine (0.54-fold), and N-acetyl serine (0.23-fold).

### 2.5. GLS2 Modulates PDH Activity and Phosphorylation Pattern

**Figure 8:**
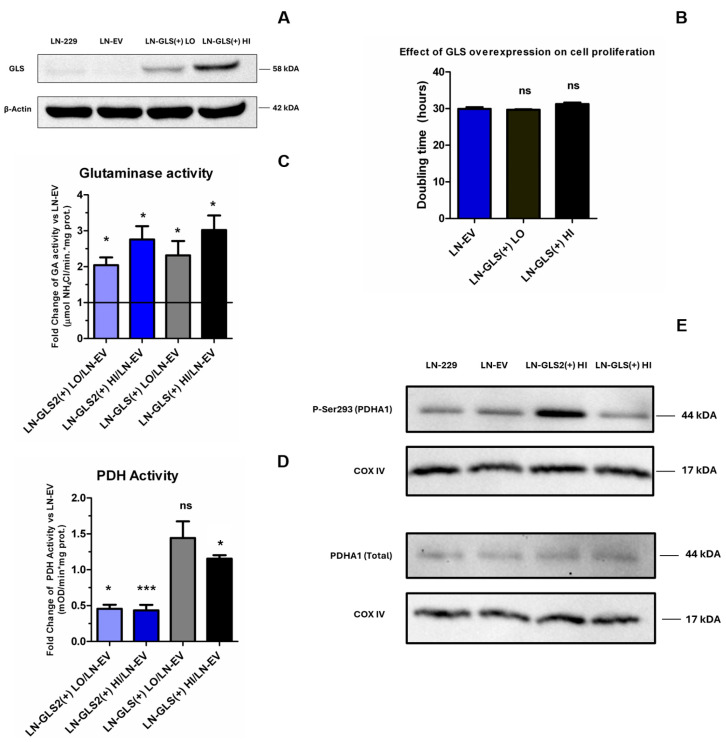
GLS2 downregulates PDH activity and induces PDHA1 phosphorylation. (**A**) Western blotting of the LN-229 models overexpressing the GLS isoform GAC for comparison with the GLS2-expressing models. Two models were selected for experiments with different levels of GLS overexpression, a lower overexpressing model (LN-GLS(+) LO) and a higher one (LN-GLS(+) HI). (**B**) Mean doubling time of the two GLS-overexpressing models compared to the LN-EV control; GLS expression did not show any significant effect on cell doubling time. (**C**) Glutaminase (GA) enzymatic activity assay showing the fold-change against the LN-EV control for normalized GA activity for LN-GLS2(+) LO, LN-GLS2(+) HI, LN-GLS(+) LO and LN-GLS(+) HI. (**D**) PDH enzymatic activity assay showing the fold-change against the LN-EV control for normalized PDH activity, for LN-GLS2(+) LO, LN-GLS2(+) HI, LN-GLS(+) LO and LN-GLS(+) HI. (**E**) Western blot images of PDHA1 phosphorylated at Ser293 and COX IV as a loading control for LN-229 (wild type), LN-EV, LN-GLS2(+) HI and LN-GLS(+) HI (top right); and of total PDHA1 protein and its COX IV loading control for LN-229 (wild type), LN-EV, LN-GLS2(+) HI and LN-GLS(+) HI (bottom right). Asterisks note the statistical significance of a *t*-test for these comparisons, as follows: *: *p*-value < 0.05; ***: *p*-value < 0.001; ns: non-significant. Western blotting experiments were repeated three times; glutaminase activity assays were repeated four times, each condition in duplicate; PDH activity was repeated three times, each condition in duplicate; and cell proliferation was assessed five times, in triplicate.

Previously shown metabolomics results pointed out that GLS2 was causing a significant drop in the 6-carbon intermediates of the TCA cycle (citrate, cis-aconitate and isocitrate), which were not being affected by concomitant CB-839 treatment. U-^13^C-Gln isotope-tracing data denoted that GLS2 was altering the way that citrate was being synthesized from Gln, as it showed a 50% reduction in citrate m + 4 abundance, while citrate m + 5 fraction was around 3-fold higher. We speculated if GLS2 could be negatively affecting PDH complex activity in an isoenzyme-specific way, which may explain lower 6-carbon intermediate levels of the TCA cycle, lower citrate m + 4 abundance, as well as pyruvate and L-alanine accumulation. We developed new LN-229 overexpression models for GLS isoenzyme to test whether an increase in the levels/activity of any GA may cause these effects, looking to compare them with the GLS2-expressing models. LN-GLS (+) models were generated similarly to LN-GLS2 (+) models, and two LN-GLS (+) with different GLS overexpression levels were selected (LN-GLS (+) LO and LN-GLS (+) HI) (Figure 8A). To test whether GLS overexpression was affecting cell proliferation, doubling time was evaluated. GLS overexpression was found to not be causing a significant change in doubling time (Figure 8B). To ascertain whether GLS2 or GLS overexpression was indeed causing an increase in GA enzymatic activity, total GA activity (i.e., GA activity due to any isoform, endogenous and/or overexpressed) was measured (Figure 8C). GA activity was increased to 2-fold for LN-GLS2(+) LO, to 2.76-fold for LN-GLS2(+) HI, to 2.31-fold for LN-GLS(+) LO and 3-fold for LN-GLS(+) HI. To analyze the potential crosstalk between GAs and PDH complex activity, a PDH enzymatic activity assay was performed (Figure 8D). Results showed that LN-GLS2(+) HI and LN-GLS2(+) LO were causing a significant reduction in PDH activity of 0.42- and 0.45-fold vs. LN-EV, respectively. LN-GLS (+) models showed no reduction in PDH activity. To further ascertain if the GLS2 effect on PDH implied the modulation of its function through phosphorylation of the catalytic subunit PDHA1, PDHA1 phosphorylation was studied for three serine residues known to downregulate PDH activity when phosphorylated: S232, S293, and S300. PDHA1 phosphorylation was analyzed by Western blotting using specific antibodies, as well as total levels of PDHA1 protein. For S232 and S300, no phosphorylation was detected in the LN-229 cell line (by comparison with the mitochondrial protein of HEK293-T cells used as a positive control). However, phosphorylated S293 of PDHA1 showed a marked increase in LN-GLS2 (+) HI (Figure 8E) compared to LN-EV and LN-229 cells. LN-GLS (+) HI did not show increased amounts of phosphorylated S293 in PDHA1. Levels of total PDHA1 protein were similar for LN-229, LN-EV, LN-GLS2(+) HI and LN-GLS (+) HI (Figure 8E).

## 3. Discussion

Cell models expressing GLS2 showed an increase in doubling times that was proportional to GLS2 expression levels (Figure 1A). Thus, GLS2 expression was able by itself to reduce the proliferation ability of these GBM cells, as it has been found for several GBM cell lines in previous studies [36,37]. However, when GLS was overexpressed in LN-229 cells, no changes in proliferation were noted (Figure 8B), so we proved that this effect was specific for GLS2, even though that overexpression of both GLS2 or GLS was causing an increase in total GA activity (Figure 8C), which was quite similar among GLS2- and GLS-overexpressing models. These results point to specific GLS2 differential functions that may or may not imply its catalytic activity. In recent years, reports have shown that GLS2 may be carrying out some activities that may not be related to its catalytic activity, being considered as potential secondary functions likely related to its protein interaction domains, as it was found to physically associate with Dicer in HCC cells to mediate the stabilization and further maturation of miR-34a, resulting in oncogenic Snail repression and an anti-metastatic effect [38]. Interestingly, GLS2 has been reported to localize into the nucleus in GBM cells [37], which may point to roles in epigenetic regulation [39]. Here, we showed that although GLS2 reduced the proliferation of GBM cells, it also made them insensitive to the anti-proliferative effects of GLS inhibition by CB-839 (Figure 1C), rendering cells resistant to CB-839 treatment (Figure 1D). It seems clear that GLS2 possesses specific functions differentiating it from GLS, and unravelling them may shed light on both its roles in physiological conditions and its impact on cancer.

In that regard, we studied how Gln carbon is handled by employing [U-^13^C]Gln when GLS2 is expressed (along with endogenous GLS expression), and also when GLS2 is expressed while CB-839 is used for GLS inhibition, an experimental condition looking to replace most of the endogenous GA activity due to GLS for GLS2 activity. GLS2 expression caused an increase in fractional abundances of succinate, fumarate, malate, and aspartate m + 4 (Figure 2A), derived from Gln through oxidative metabolism in the TCA cycle of Gln-derived AKG [35], which was expected since these models have higher global GA activity. Accordingly, when CB-839 was added, fractional abundances of these metabolites tended to return to the control levels in the lower GLS2-expressing model (Figure 1B). In contrast, citrate m + 4, which is a product of oxidative Gln-derived oxaloacetate condensation with pyruvate-derived acetyl-CoA, was reduced when GLS2 was highly expressed (Figure 1A), as citrate m + 4 fractional abundance was reduced by 50%, not being affected by CB-839 treatment (Figure 1B). All of this pointed to GLS2 causing a blockade in citrate synthesis through oxidative TCA cycle metabolism, which was not merely caused by an increase in GA activity, since GLS inhibition by CB-839 did not modify citrate m + 4 abundance. On the other hand, GLS2 expression increased m + 5 citrate (Figure 3A) from reductive carboxylation of Gln-derived AKG, and also m + 3 aspartate, malate and fumarate, which are derived from citrate m + 5 [35].

Metabolomic quantification of TCA cycle-related metabolites (Figure 6) showed lower levels of Gln, and higher levels of glutamate, AKG, succinate, fumarate, malate and aspartate when GLS2 was expressed at high levels (Figure 6A), while the 6-carbon intermediates of the TCA cycle, citrate, cis-aconitate and isocitrate, were largely reduced. LN-GLS2(+) LO, showed lower Gln, and higher glutamate, succinate and aspartate, but again lower levels of citrate, cis-aconitate and isocitrate (Figure 6B). Integration of both isotope-tracing and metabolomics suggests that GLS2 expression increases the metabolic flux from Gln to the TCA cycle, increasing the levels of most metabolites of the cycle, but at the same time causing a blockade in the oxidative synthesis of citrate, denoted by a reduction in citrate m + 4, and global citrate levels (Figure 2A and Figure 6A,B, respectively), while citrate m + 5 increase (Figure 3A) may be a consequence of both the hypothetical blockade in oxidative citrate synthesis and increased influx via glutaminolysis into the TCA cycle. Interestingly, when the lower GLS2-overexpressing model was treated with CB-839 (Figure 6D), levels of Gln, glutamate, succinate and aspartate returned to the control levels, while citrate, cis-aconitate and isocitrate were not affected, showing the same differences as the untreated model (Figure 6B). Again, all this suggested that the effect of GLS2 on citrate synthesis, and subsequent synthesis of cis-aconitate and isocitrate, is specific for GLS2 and not due to any change in GA activity.

Additionally, LN-GLS2(+) HI also showed an accumulation of pyruvate (Figure 7), the substrate of PDH for acetyl-CoA generation in the mitochondria. Acetyl-CoA is a substrate-needed reaction with oxaloacetate to form citrate in the TCA cycle. In this regard, it appears logical to consider that the GLS2-induced blockade of citrate synthesis could be mediated by a negative modulatory effect of GLS2 on PDH enzymatic activity that would impair acetyl-CoA production, thus making it limiting for citrate synthesis. Acetyl-carnitine was reduced by 50% with high GLS2 expression (Figure 7). We hypothesize that altered acetyl-carnitine levels may be reflecting changes in acetyl-CoA levels, as we have discussed in a previous publication [35]. Acetyl-carnitine takes part in an interchange reaction catalyzed by carnitine acetyltransferase with acetyl-CoA, for the transfer of the acetyl group to carnitine to be translocated though cell membranes [40], which is essential in some situations such as when the TCA cycle flux is compromised and acetyl-CoA accumulates and sequesters the mitochondrial pool of HS-CoA, preventing it from participating in other reactions [41]. In addition, the levels of several N-acetyl amino acids significantly changed with GLS2 overexpression (Appendix D), and though they can be synthesized in different ways, acetyl-CoA is always the acetyl donor for the acetylation reaction [42]. We hypothesize that the generalized diminished levels of N-acetyl amino acids may also be indicative of lower acetyl-CoA levels, that may be limiting for citrate synthesis in the TCA cycle.

To ascertain the potential crosstalk between GLS2 and PDH, which may explain the previous metabolic changes, PDH activity was assessed. To further confirm if this potential effect on PDH was specific for GLS2, and not for other glutaminases, two cell models were developed to overexpress GLS, at lower (LN-GLS(+) LO) and higher (LN-GLS(+) HI) levels (Figure 8A). Both GLS2- and GLS-overexpressing models showed an increase in total GA activity (Figure 8C). PDH activity was greatly decreased when GLS2 was overexpressed in both HI and LO models by more than 55% (Figure 8D). On the other hand, GLS overexpression did not cause lower PDH activity. To further deepen the mechanism by which GLS2 caused lower PDH activity, the phosphorylation status of three serine residues of PDHA1 was analyzed by Western blotting. Serine 293, serine 300, and serine 232 are known to be phosphorylated by pyruvate dehydrogenase kinases 1-4 (PDK1-4) to negatively regulate PDH activity [43,44]. Phosphorylated serine 232 and serine 300 of PDHA1 were not detected in the LN-229 cell line; however, phosphorylated serine 293 was found to be significantly increased when GLS2 was overexpressed (Figure 8E), while no changes were noted when GLS was being overexpressed. Total PDHA1 levels were analyzed in parallel to confirm that variations in PDH activity and/or PDHA1 phosphorylation were not due to changes in protein levels. No significant alterations in PDHA1 levels were noted in any condition (Figure 8E). Considering all of the above, GLS2 was found to be specifically causing an increase in phosphorylation of serine 293 of PDHA1, causing PDH activity to decrease, which could be preventing glycolytic carbon from being incorporated into mitochondrial metabolism, minimizing the metabolic flux through the full TCA cycle and likely negatively affecting the OXPHOS rate, which may be related to the lower proliferation capacity induced by GLS2 (Figure 1B). Mitochondrial metabolism has been classically considered to be impaired in cancer [45]; however, it is nowadays evident that the picture is more complex and that most cancer cells and tumours need and rely on mitochondrial metabolism [3]. Notably, the metabolic changes induced by GLS2 did not correlate with higher lactate levels (Figure 7), so although GLS2 could be limiting glycolytic flux into the mitochondria, it did not cause an increase in the Warburg effect (increased aerobic glycolysis and lactate generation).

Further research is needed to investigate the mechanism by which GLS2 induces PDHA1 phosphorylation and, hence, lower PDH complex activity. The PDH complex can be regulated at several levels and by various substrates [46]. However, phosphorylation of PDHA1 at serine residues 232, 293, and 300 by PDKs 1-4 are well studied and established regulation mechanisms, while PDH phosphatases 1 and 2 mediate in its dephosphorylation and activation [47,48,49]. Phosphorylation of one of the three serine residues is enough to inhibit PDH complex activity [50,51]. It seems reasonable to think that GLS2 could be inducing one or more PDKs to increase the phosphorylation of S293 in PDHA1. Whether this inhibition of PDH activity by GLS2 is a generalizable characteristic of GLS2 in other tumour types or even in physiological conditions is another key question to be addressed. GLS2 was originally discovered in the liver [52], and its expression is restricted to certain tissues/organs, like the brain, pancreas, or liver, where it is highly expressed [53]. Given the fact that the liver is a gluconeogenic organ, it is tempting to speculate that PDH activity regulation by GLS2 could be related to GLS2’s homeostatic functions in normal physiological conditions in the liver. In this regard, GLS2 could be a more suitable GA isoenzyme for adapting to a gluconeogenic metabolism, favouring glutaminolysis while preventing pyruvate funnelling toward the TCA cycle. In this hypothetical scheme, GLS2 could be providing gluconeogenic substrates by glutaminolysis such as oxaloacetate, which can be a substrate of phosphoenolpyruvate carboxykinase (PEPCK) to produce the glycolytic intermediate phosphoenol pyruvate [54], while concomitantly inhibiting PDH function, and thus preventing both pyruvate utilization by PDH and further oxaloacetate condensation with PDH-derived acetyl-CoA. In this case, GLS2 could then be promoting a net directionality towards gluconeogenesis and glucose synthesis. However, although this hypothetical role of GLS2 isoenzyme fits well with its preponderant expression in the liver, this currently only lies on the grounds of speculation, and further efforts are needed to ascertain the role of GLS2 both in physiological conditions and in cancer.

Moreover, GLS2 expression affected the levels of several nucleotides (Appendix B). AMP was accumulated in both GLS2-overexpressing models, treated or not treated with CB-839, while GMP and UMP were reduced in LN-GLS2(+) HI, and also in both GLS2-overexpressing models when CB-839 was added (Appendix B). These changes may be a consequence of altered de novo nucleotide biosynthesis; however, lower levels of GMP and UMP with GLS2 expression were unanticipated since GLS2 caused aspartate accumulation (Figure 6), which is needed for purine and pyrimidine biosynthesis. Another interpretation implies the effect of GLS2 on PDH and subsequent lower TCA cycle activity, which would minimize NADH formation, OXPHOS rate and ATP synthesis, resulting in a higher AMP/ATP ratio and, therefore, AMP accumulation. ATP is needed for nucleotide biosynthesis and its hypothetical shortage may also explain lower levels of GMP.

**Figure A3:**
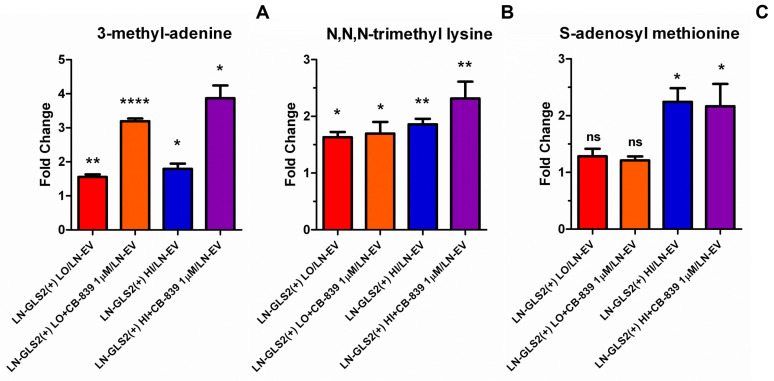
GLS2 induces changes in methylation-related metabolites. Changes in the levels of the methylated metabolites 3-methyl-adenine (**A**) and N,N,N-trimethyl-lysine (**B**), and the methyl donor metabolite S-adenosyl-methionine (SAM) (**C**) for LN-GLS2(+) LO, LN-GLS2(+) LO +CB- 839 1 µM, LN-GLS2(+) HI and LN-GLS2(+) HI +CB-839 1 µM, expressed as the fold-change against the LN-EV control. Asterisks note the statistical significance of a *t*-test for these comparisons, as follows: *: *p*-value < 0.05; **: *p*-value < 0.01; ****: *p*-value < 0.0001; ns: non-significant.

Lastly, GLS2 caused an increase in the methylated metabolites 3-methyl-adenine (3-MA) (Appendix C, Figure A3A) and N,N,N-trimethyl lysine (3meLys) (Figure A3B). As previously discussed by authors [35], 3-MA may appear as a spontaneous modification of DNA to be repaired by alkyl-DNA glycosylases, through the base excision repair mechanism, releasing free 3-MA [55].

On the other hand, multiple lysine residues in histones are targets for methylation by lysine methyltransferases, with essential roles in epigenetic regulation [56]. Proteolytic degradation of methylated histones has been identified as the primary source of free 3meLys. Free 3meLys is implicated in the carnitine biosynthesis pathway, where AKG acts as a cosubstrate, being oxidized to succinate [57]. Since AKG is higher when GLS2 is overexpressed, carnitine synthesis does not seem to be impaired. Thus, free 3-MA and 3meLys could be interpreted as indicators of overall methylation levels in DNA and proteins. Interestingly, concomitant treatment with CB-839 further increased 3-MA (Figure A3A) and 3meLys’ levels (Figure A3B).

GLS2 expression has been reported to downregulate the phosphatidylinositol 3-kinase/protein kinase B (PI3K/AKT) signalling pathway in HCC [58] and GBM [36] cell lines, which has been related to a tumour suppressor function for GLS2 [37,59,60]. Interestingly, inhibition of the PI3K-AKT pathway has been shown to regulate oxygen metabolism in human head and neck cancer cells through a mechanism involving phosphorylation of PDH [61]. In addition, 3-MA has been used as an autophagy inhibitor through downregulation of the PI3K/AKT pathway [62]. Although further research is needed to confirm if endogenous levels of 3-MA can act in this sense, speculations can be made linking increased 3-MA when GLS2 is overexpressed and previously noted downregulation of the PI3K/AKT pathway related to GLS2 expression. In this regard, and considering previous reports on head and neck cancer cells [61], it may be hypothesized that the effect of GLS2 on the PI3K/AKT pathway may be related to our finding linking GLS2 and PDH activity downregulation.

Noticeably, SAM levels were higher in the LN-GLS2(+) HI model (Figure A3C) and this change was not affected by treatment with CB-839, pointing to a specific effect of GLS2. We have shown previously that CB-839 may causes 3-MA, 3meLys and also 5-methyl cytosine levels to increase in GBM cells, which was thought to be due to imbalanced levels of AKG and succinate, implying AKG dioxygenases [35] GLS2 did not cause relative imbalance between AKG and succinate, so the potential effect of GLS2 on overall methylation levels is thought to be mediated by increased SAM levels, which is a critical methyl donor for methylation reactions [63]. However, the mechanism by which GLS2 could be causing an accumulation of SAM remains to be elucidated.

Future research is needed to ascertain how generalizable the molecular and metabolic changes induced by GLS2 are, as well as the mechanisms behind them, which could imply (or not imply) the catalytic GA activity of GLS2, its interactome, or its subcellular location. Previous publications by our group found that GLS2 could be located at the nucleus of GBM cells [37], as well as linking GLS2 function with oxidative stress-handling [58] and microRNA regulation [59], also noted previously in HCC by other groups [38], while also reporting modulation of cell signalling pathways by GLS2 in HCC and GBM [36,57]. Despite all of the above, it is not to be ruled out that the GLS2-PDH crosstalk and the effect on the TCA cycle, nucleotide biosynthesis, or methylation modulation may be a consequence of epigenetic and/or protein function regulation at different levels carried out by GLS2. Understanding GLS2 functions will shed light on its role in physiological conditions and in cancer, where the aim is to identify GLS2 expression as an indicator of certain molecular vulnerabilities to be exploited in future targeted therapies.

## 4. Materials and Methods

### 4.1. Chemicals

Dimethyl sulfoxide (DMSO), CB-839 (Telaglenastat), methoxyamine hydrochloride, pyridine, tert-butyldimethylsilyl ether (TBDMS) and G418 were obtained from Sigma-Aldrich Co. (St. Louis, MO, USA). U-^13^C-Gln was purchased from Cambridge Isotope Laboratories (Tewksbury, MA, USA). Hoechst and propidium iodide were from ThermoFisher Scientific (Waltham, MA, USA).

### 4.2. Cell Lines, Culture Conditions and Stable Transfections

The LN-229 human GBM cell line was kindly provided by Dr. Monika Szeliga, Department of Neurotoxicology, Mossakowski Medical Research Centre, Polish Academy of Sciences, Warsaw, Poland. Cells were cultured in Dulbecco’s Modified Eagle Medium (DMEM) supplemented with 10% foetal bovine serum, 100 I.U./mL penicillin, 100 μg/mL streptomycin, and 4 mM L-Gln, as previously described by authors [59]. For stable overexpression experiments, LN-229 cells were seeded in 6-well plates and transfected with a pcDNA3 vector (GenScript Biotech, Piscataway, NJ, USA) containing the Neomycin resistance gene and the full-length cDNA of GLS2 (for the GLS2-overexpressing models), the full-length cDNA of the GAC isoform of GLS (for the GLS-overexpressing models), or the empty vector (for control cells), employing Lipofectamine 3000 (ThermoFisher Scientific). Transfected cell pools were incubated for 48 h and then supplemented with 0.75 mg/mL G418 (Sigma-Aldrich, St. Louis, MO, USA) for the selection of transfected cells. Cell media supplemented with G418 was renewed every two days for 7–10 days to allow for the selection and limited expansion of transfectants (not allowing cells to reach high confluence percentages). After this time, cells were detached, and individual cells were isolated by seeding them in 96-well plates by limiting dilution cloning. Wells were checked subsequently to contain individual cells and wells containing no cells or more than one cell were discarded. Monoclonal populations were expanded for 4–6 weeks using media supplemented with G418. Total protein was extracted from each monoclonal population generated to verify the expression of the protein of interest (GLS2 or GLS) by Western blotting using specific antibodies. Two independent cell models were selected for each condition (overexpression of GLS2 or GLS), having different expression levels of the protein of interest. Cells were transfected with the empty vector following the same procedure as the overexpressing models and were employed as controls. LN229-GLS2(+) (including the lower GLS2-expressing model called LN-GLS2(+) LO and the higher GLS2-expressing model called LN-GLS2(+) HI), empty vector-transfected cells LN229-EV (named LN-EV) and LN229-GLS(+) (including the lower GLS-expressing model called LN-GLS(+) LO and the higher GLS-expressing model called LN-GLS(+) HI) were maintained in DMEM routinely supplemented with 0.75 mg/mL G418 for continuous selection of stable transfectants, preventing spontaneous loss of expression of the protein of interest. Stable overexpression of GLS2 or GLS was routinely checked by Western blotting, proving stable expression over time. All cultures were maintained at 37 °C in a humidified atmosphere with 95% air and 5% CO_2_. Cells were routinely checked to be free of mycoplasma contamination.

### 4.3. Cell Proliferation and Viability Assays

For studying changes in cell proliferation, 4 × 10^4^ cells of each experimental or control (LN-EV) condition were seeded in triplicate in 12-well plates. Cells were allowed to grow for 96 h and then detached and counted using trypan blue as vital staining for discriminating live or dead cells. A total of 4 × 10^4^ cells of each replicate were independently reseeded, and the process was repeated for 5 cycles, so each replicate of each condition grew independently for a total of 20 days. The cell number in each count was referred to initial seeding number to calculate a mean doubling time for each condition.

To analyze cell viability, 2.5 × 10^3^ cells for each cell model were seeded in 96-well plates. CB-839 was added in a concentration range from 0.01 nM to 10 µM. DMSO was used as a vehicle control to compare with, considered as the 100% viability condition. Each condition was assayed in quadruplicate. After 72 h of treatment, the culture medium was carefully replaced with medium supplemented with Hoechst at 1 µg/mL and propidium iodide at 1 µg/mL (ThermoFisher Scientific, Waltham, MA, USA) for total and dead cell staining, respectively. The plate was covered and incubated at 37 °C for 30 min and was then analyzed in a Celigo Image Cytometer (Nexcelom Bioscience, Lawrence, MA, USA). The viable cell number was calculated for each condition and a viability ratio was calculated by considering the DMSO-treated cells as the 100% viability (viability = 1). Inhibitory concentration 50 (IC50) (viability ratio = 0.5) was calculated, when possible, by referring the viability ratio to the CB-839 concentration.

### 4.4. Metabolomics

For the extraction of the cell metabolome, samples were processed as previously described [35]. Briefly, cells were cultured in 6 cm dishes, and when they reached 80–90% confluence, the cells were washed with ice-cold normal saline solution on ice, and then 1 mL of −20 °C cold 80% *v*/*v* methanol was added per dish. The cells were scraped on ice, and the suspension was transferred to microcentrifuge tubes and subjected to three freeze–thaw cycles between liquid nitrogen and a 37 °C water bath. Lysates were centrifuged at 4 °C for 15 min at 17,000× *g*, and the metabolite-containing supernatant was transferred to new microcentrifuge tubes and dried overnight in a Speed-Vac concentrator (Thermo Fisher Scientific, Waltham, MA, USA, SPD1030), keeping the tubes open and applying breathable membranes to avoid cross-contamination. Metabolite-containing pellets were redissolved and transferred to liquid chromatography–mass spectrometry (LC–MS) autosampler vials and injected on a Quadrupole Time of Flight (QTOF) LC–MS analyser (Agilent Technologies, 6550 iFunnel, Santa Clara, CA, USA). The total ion count for each sample was employed to normalize metabolite abundances, and a fold-change was calculated by referring experimental normalized abundances to the control ones.

### 4.5. [U-^13^C] Glutamine Tracing Experiments

Cells were cultured with or without CB-839 and when they reached 80% confluence, cells were washed with PBS twice, and fresh medium containing labelled Gln with or without CB-839 treatment was added to each dish. Then, cells were traced for 6 h, washed with ice-cold normal saline, and metabolites were extracted in methanol and processed as described before. Dried metabolite pellets were incubated with 1% methoxyamine hydrochloride in pyridine for 15 min at 70 °C, following derivatization with TBDMS for 1 h at 70 °C. The volume was transferred to gas chromatography-mass spectrometry (GC-MS) autosampler vials, and 1 µL was injected into an Agilent 7890A gas chromatograph coupled to an Agilent 5975C mass selective detector (Agilent Technologies, Santa Clara, CA, USA). The results were manually reviewed, and data were expressed as the isotopologue fractions of the total metabolite.

### 4.6. Protein Expression and Mitochondria Isolation

GLS2 or GLS (GAC isoform) overexpression was assessed by Western blotting (Figure 1A and Figure 8A). Cells were washed in PBS and harvested in lysis buffer (RIPA buffer: 1% NP-40, 0.1% sodium deoxycholate, 0.1% SDS, 150 mM NaCl, 50 mM Tris-HCl pH 8.0, supplemented with EDTA-free Halt™ (100×) Protease Inhibitor Cocktail, (ThermoFisher Scientific, Waltham, MA, USA). Lysates were vortexed, incubated on ice for 5 min, and then centrifuged at 12,000× *g*, 15 min at 4 °C. Supernatants were collected and assayed or stored at −80 °C. Protein quantification was performed using the Pierce™ BCA Protein Assay Kit (ThermoFisher Scientific, Waltham, MA, USA). Twenty micrograms of total protein were resolved on 10% polyacrylamide-SDS gels and then electrotransferred to nitrocellulose membranes. Membranes were blocked for 1 h at room temperature with 5% non-fat milk or bovine serum albumin (BSA) in Tris-buffered saline-Tween 20 (TBS-T). All antibodies were diluted in 5% BSA in TBS-T. For GLS detection, a primary antibody targeting specifically the GAC isoform was used. This antibody was generated from rabbits against a peptide containing the 20 C-terminal amino acids of human GAC, and has been previously demonstrated to react specifically with the GAC isoform of GLS, and not with other glutaminase isoforms [59]. For GLS2 quantification, an antibody against GLS2 (ab113509) was purchased from Abcam (Abcam, Cambridge, UK), and used at 1:2000 dilution. An antibody targeting β-actin (ab8227, Abcam, Cambridge, UK) was used at 1:5000 for employing the β-actin signal as a loading control. To analyze pyruvate dehydrogenase E1 subunit alpha 1 (PDHA1) levels and its phosphorylation status, mitochondria were isolated from cells in culture using the mitochondria isolation kit for cultured cells (89,874, ThermoFisher Scientific, Waltham, MA, USA). Isolated mitochondria were lysed with RIPA buffer supplemented with protease (87,785, ThermoFisher Scientific, Waltham, MA, USA) and phosphatase inhibitors (78,420, ThermoFisher Scientific, Waltham, MA, USA), and mitochondrial protein was quantified using the Pierce™ BCA Protein Assay Kit. A total of 15 µg of mitochondrial proteins were loaded and resolved on 15% polyacrylamide-SDS gels and electrotransferred to PVDF membranes. Membranes were blocked for 1.5 h at room temperature with 5% BSA in TBS-T. PDHA1 phosphorylation was studied at three serine residues (AP1063, Sigma-Aldrich, St. Louis, MO, USA, for p-Ser232, 1:500 dilution; ab177461, Abcam, Cambridge, UK, for p-Ser293, 1:1000 dilution; and 29583-1-AP, ThermoFisher Scientific, Waltham, MA, USA, for p-Ser300, 1:1000 dilution), as well as total PDHA1 (ab168379, Abcam, Cambridge, UK, 1:1000 dilution). COX IV was used as a loading control for mitochondrial protein, using a specific antibody (ab16056, Abcam, Cambridge, UK, 1:5000 dilution). All incubations with primary antibodies were performed overnight at 4 °C in agitation. After incubation with respective primary antibodies, the membranes were washed in TBS-T and incubated for 1 h at room temperature with a secondary HRP-conjugated Goat anti-rabbit antibody (A0545, Sigma-Aldrich, St. Louis, MO, USA, 1:40,000 dilution) and visualized in a ChemiDoc™ Gel Imaging System (Bio-Rad, Hercules, CA, USA), using an enhanced chemiluminescence detection system (SuperSignal™ West Pico, ThermoFisher Scientific, Waltham, MA, USA). For data quantification in the case of PDHA1/COXIV Western blots, Image Lab Software v5.2. (Bio-Rad) was used, following the manufacturer’s instructions. In brief, each band was detected and selected, and the background signal was discarded. After that, net values corresponding to the signal of the protein of interest (phosphorylated or total PDHA1) band were normalized to their respective loading control (COX IV) signal (Appendix A).

### 4.7. Glutaminase Enzymatic Activity

Glutaminase activity was estimated in the experimental models overexpressing GLS2 (LN-GLS2(+) LO and LN-GLS2(+) HI) or GLS (LN-GLS(+) LO and LN-GLS(+) HI) by quantification of the ammonium produced in the GA reaction. The method was adapted from the original method described by McGee and Knox [64] and modified by Heini et al. [65], based on the reaction of ammonium and o-phthalaldehyde (OPA), which may be further quantified by spectrophotometry or fluorescence. In brief, about 4 × 10^6^ cells of each condition were cultured and detached using trypsin when it reached 80% confluence. Cells were centrifuged and washed twice with PBS, then cell pellets were resuspended in 300 µL of 150 mM K_2_HPO_4_, pH 8.6. Samples were subjected to two freeze–thaw cycles and vortexed for 1 min to completely break cell membranes. Samples were centrifuged at 12,000× *g*, 5 min at 4 °C and supernatant was collected. Total protein was quantified using the Pierce™ BCA Protein Assay Kit (ThermoFisher Scientific, Waltham, MA, USA) for further normalization. A total of 40 µL of extracts were loaded in 96-well plates, in triplicate, and 56 µL of reaction buffer (171 mM L-Gln, 150 mM K_2_HPO_4_, pH 8.6) was added and mixed. Plates were incubated for 1 h at 37 °C and then the reaction was stopped by adding 10 µL of 10% *v*/*v* trichloroacetic acid. Blanks were included for each sample by incubating sample extracts and reaction buffer separately and mixing just before trichloroacetic acid addition. For quantification of the ammonia generated in the GA reaction, 30 µL of each reaction was transferred to new wells and 150 µL of the OPA reagent (8.6 mM OPA, 3.33 mM β-mercaptoethanol, 160 mM K_2_HPO_4_, pH 7.4) was added. A standard curve of NH_4_Cl ranging from 2.5 mM to 75 µM was assayed in parallel. The plate was covered and incubated for 45 min, at 37 °C in the dark, and then absorbance was measured at 410 nm in a plate reader. The absorbance of the blanks was subtracted from the samples to account for ammonia generation that not due to GA reaction, and net absorbance values were used to calculate GA activity by referring them to the NH_4_Cl standard curve. Values were normalized to total protein content and referred to the control to calculate a fold-change. Four independent experiments were performed.

### 4.8. PDH Enzymatic Activity

PDH activity was quantified using the Pyruvate Dehydrogenase (PDH) Enzyme Activity Microplate Assay Kit (ab109902, Abcam, Cambridge, UK). Samples were processed and assayed according to the manufacturer’s instructions. Briefly, cells were cultured in 150 mm dishes, employing at least 2 × 10^7^ cells per condition. When cells reached 80% confluence, the medium was discarded, and dishes were washed twice with ice-cold PBS on ice. Cells were scraped in ice-cold PBS and samples were transferred to microcentrifuge tubes. Cells were centrifuged at 300× *g*, for 5 min at 4 °C, and the cell pellet was resuspended in 500 µL ice-cold PBS supplemented with phosphatase inhibitors. A small aliquot was taken for protein quantification, and the cell suspension was accordingly adjusted to the equivalent of 15 mg/mL of protein. After that, detergent was added for cell lysis, and lysates were centrifuged at 1000× *g*, for 10 min a 4 °C, transferring supernatant to clean tubes. The extract was diluted to 2–5 mg/mL of protein and 200 µL was loaded in 96-well plates functionalized with monoclonal antibodies against PDH for intact PDH complex immobilization. Samples were incubated for 3 h at room temperature to allow for binding of the PDH complex. After that, the volume was discarded and wells were washed twice; then, a substrate/chromogen solution was added, and the plate was kinetically read at 450 nm in a plate reader for 30 min. Data were analyzed according to the manufacturer’s instructions, by comparing absorbance increase rate between experimental and control samples, normalized to protein content.

### 4.9. Statistical Analysis

Unless specifically noted otherwise, at least three independent experiments were performed in duplicate; for tracing experiments, three independent experiments were made in triplicate; and metabolomics was made twice in triplicate. Heatmaps showing the change in abundance of the top 50 altered metabolites were generated by using the web tool Metaboanalyst, following PLS-DA analysis. Dot and bar graphs and statistical analysis (Student’s *t*-test for experimental against control samples) were made using GraphPad Prism software v6. Values were expressed as means ± standard error of the mean (SEM). *p <* 0.05 was considered statistically significant. Statistical significance was expressed using asterisks, as follows: * *p <* 0.05, ** *p <* 0.01, *** *p <* 0.001, **** *p <* 0.0001.

## 5. Conclusions

This study focused on the metabolic changes induced by ectopic GLS2 expression in GBM cells, which are characterized by high levels of GLS but lack appreciable GLS2 expression [25]. We have identified for the first time specific crosstalk between GLS2 and PDH, placing GLS2 as a potential key regulator of mitochondrial metabolism, linking glucose and glutamine metabolism, essential metabolic pathways for cancer cells [66]. This finding may explain some of the tumour suppressor functions associated with GLS2 in several contexts, including GBM models [11,36]. Additionally, GLS2 induced accumulation of AMP, and depletion of GMP and UMP, likely reflecting alterations in nucleotide biosynthesis and bioenergetics, which could not be compensated through inhibition of endogenous GLS by CB-839, even when aspartate and glutamine overall levels where not altered. In addition, GLS2 induced an accumulation of methylated metabolites, potentially indicating higher overall methylation in DNA and proteins, including histones, which was further increased by concomitant GLS inhibition, reflecting the opposite effects of GLS and GLS2. GLS2 induced an accumulation of SAM that was not affected by GLS inhibition, which appears as the mechanistic reason for increased hypothetical methylation. However, the mechanism by which GLS2 may be increasing SAM levels, downregulating GMP and UMP, and increasing phosphorylation of serine 293 of PDHA1 remains unknown. Our findings paved the way for further research regarding deeper mechanistic studies concerning the implication of GLS2 in PDH modulation, nucleotide metabolism and epigenetic regulation. Whether these activities for GLS2 take place in other tumour types, or are even related to the role of GLS2 in normal physiological conditions, remains a key open question to be solved.

## Figures and Tables

**Figure A1:**
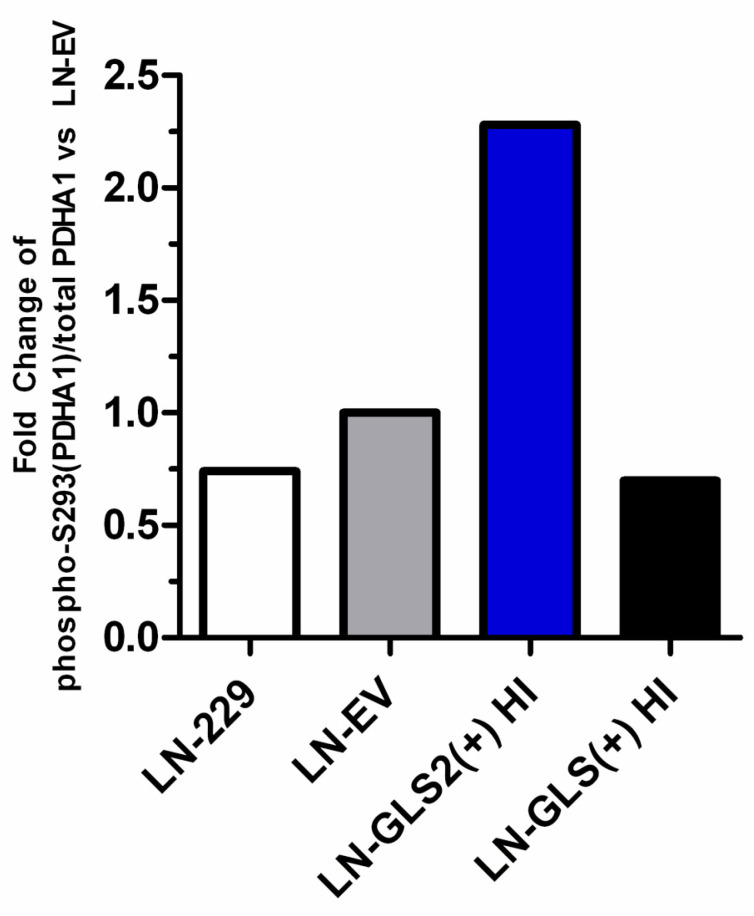
Western blot quantification for phosphorylated PDHA1 at S293 and total PDHA1. Phospho-S293 signal (image shown in Figure 8E) was quantified, and values were normalized to COXIV signal for each sample. The same procedure was followed for total PDHA1 levels; then, normalized phospho-S293 values were referred to normalized total PDHA1 values. For clearer interpretation, data are expressed as a fold-change by relativizing phospho-S293 PDHA1/total PDHA1 values to the LN-EV control.

**Figure A2:**
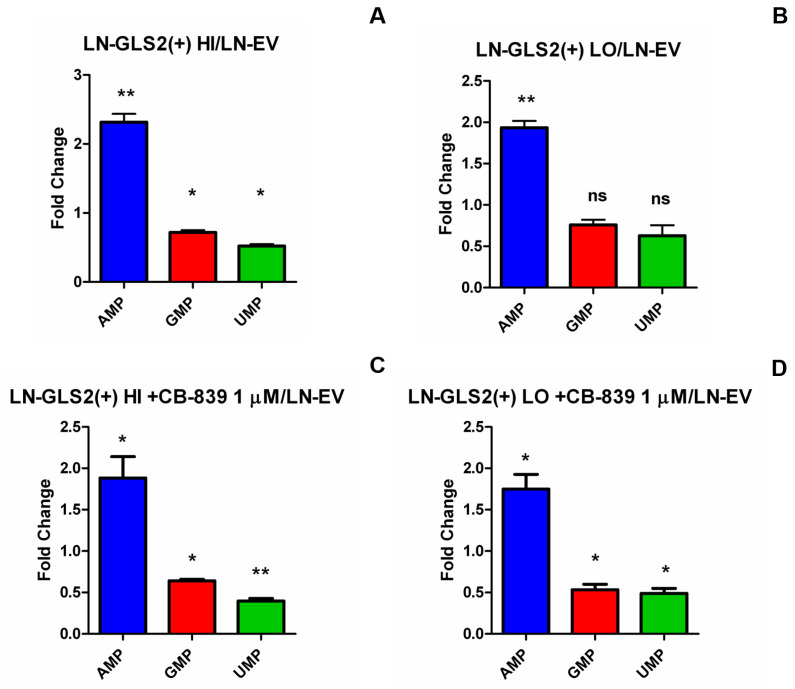
GLS2 alters nucleotide levels. Changes in the levels of adenosine monophosphate (AMP), guanosine monophosphate (GMP) and uridine monophosphate (UMP) expressed as the fold-change compared to the control (LN-EV) in the LN-GLS2(+) HI model (**A**), LN-GLS2(+) HI +CB-839 1 µM (**B**), LN-GLS2(+) LO (**C**), and LN-GLS2(+) LO +CB-839 1 µM (**D**). Asterisks note the statistical significance of a *t*-test for these comparisons, as follows: *: *p*-value < 0.05; **: *p*-value < 0.01; ns: non-significant.

**Figure A4:**
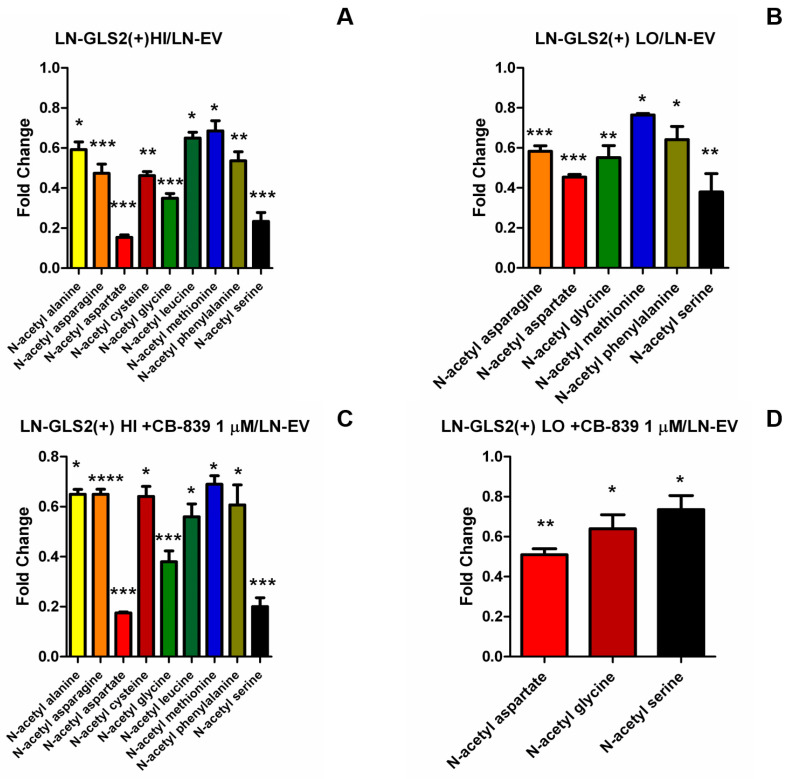
GLS2 alters the levels of N-acetyl amino acids. Fold-change in the levels of several N-acetyl amino acids comparing LN-GLS2(+) HI vs. LN-EV (**A**), LN-GLS2(+) LO vs. LN-EV (**B**), LN-GLS2(+) HI + CB-839 1 µM vs. LN-EV (**C**), or LN-GLS2(+) LO + CB-839 1 µM vs. LN-EV (**D**). Only significantly altered N-acetyl amino acids are shown. Asterisks note the statistical significance of a *t*-test for these comparisons, as follows: *: *p*-value < 0.05; **: *p*-value < 0.01; ***: *p*-value < 0.001; ****: *p*-value < 0.0001.

## Data Availability

All data generated in this study are available from the corresponding authors upon reasonable request.
